# Efficacy of the Benznidazole+Posaconazole combination therapy in parasitemia reduction: An experimental murine model of acute Chagas

**DOI:** 10.1590/0037-8682-0477-2019

**Published:** 2020-02-07

**Authors:** Luis Eduardo Echeverría, Clara Isabel González, Julio Cesar Mantilla Hernandez, Martha Lucia Díaz, Javier Eduardo, Luis Alberto López-Romero, Julián David Rivera, Edwin Uriel Suárez, Sergio Alejandro Gómez Ochoa, Lyda Z. Rojas, Carlos A. Morillo

**Affiliations:** 1 Grupo de Estudios Epidemiológicos y Salud Pública-FCV, Fundación Cardiovascular de Colombia, Floridablanca, Colombia.; 2 Heart Failure and Heart Transplant Clinic, Fundación Cardiovascular de Colombia, Floridablanca, Colombia.; 3 Basic Sciences Department, Faculty of Medicine. Universidad Industrial de Santander. Grupo de Inmunología y Epidemiología Molecular GIEM, Santander, Bucaramanga, Colombia.; 4 Veterinary Department. Universidad Cooperativa de Colombia, Bucaramanga, Santander, Colombia.; 5 Research Group and Development of Nursing Knowledge (GIDCEN-FCV), Research Institute, Fundación Cardiovascular de Colombia, Floridablanca, Santander, Colombia.; 6 Division of Cardiology, Department of Cardiac Sciences, Libin Cardiovascular Institute of Alberta, University of Calgary, Alberta, Canada.; 7 Department of Medicine, Cardiology Division, McMaster University, PHRI-HHSC, Hamilton, Ontario, Canada.

**Keywords:** Wistar rats, Chagas Disease, Trypanocidal, Benznidazole and Posaconazole

## Abstract

**INTRODUCTION:**

Benznidazole (BZL) and Nifurtimox (NFX) are the pharmacological treatment for acute phase Chagas Disease (CD); however, therapy resistance and residual mortality development remain important unresolved issues. Posaconazole (POS) has shown a trypanocidal effect in vivo and in vitro. Thus, this study aimed at comparing the *T. Cruzi* parasitic load-reducing effect of the combination of BZL+POS against that of monotherapy with either, during acute phase CD, in an experimental murine model.

**METHODS:**

Nineteen *Wistar rats* were randomly allocated to four groups and inoculated with the trypomastigotes of *T. cruzi* strain´s JChVcl1. The rats were administered anti-parasites from day 20-29 post-infection. The Pizzi and Brener method was used for parasitemia measurement. Longitudinal data analysis for the continuous outcome of repeated measures was performed using parasitemia as the outcome measured at days 20, 22, 24, 27, and 29 post-infection.

**RESULTS:**

All four groups had similar parasitic loads (p=0.143) prior to therapy initiation. Among the three treatment groups, the BZL+POS (n=5) group showed the highest mean parasitic load reduction (p=0.000) compared with the control group. Likewise, the BZL+POS group rats showed an earlier therapeutic effect and were the only ones without parasites in their myocardial samples.

**CONCLUSIONS:**

Treatment of acute phase CD with BZL+POS was more efficacious at parasitemia and myocardial injury reduction, compared with monotherapy with either.

## INTRODUCTION

Chagas Disease (CD) is currently a global public health problem. According to the World Health Organization (WHO), approximately 6-7 million people are infected, and 110 million people are at risk of infection^1^ ^,^ ^2^. About 12.500 deaths/year in Latin America are attributed to CD^3^ ^,^ ^4^. Colombia has approximately 0.7-1.2 million infected inhabitants and 8 million persons at risk of infection^4^ ^,^ ^5^. In 2017, 473 new cases of CD were reported; 19 acute CD cases^6^. Additionally, from 2008-2012, there were six acute Chagas outbreaks, including three with the most fatal outcomes^7^ ^,^ ^8^.

CD has two consecutive phases: acute and chronic. The acute phase is often asymptomatic, with most patients recovering with no damage to target organs such as the heart, esophagus, and colon. Almost half of CD patients remain in this indeterminate form, while the other half develop chronic forms of the disease with cardiac or digestive damage, 10-30 years after the initial infection^2^ ^,^ ^9^.

The acute phase is mostly treated with anti-parasites and heart failure medication, in cases with acute myocarditis^10^. In the last 50 years, only two anti-parasitic medications have been approved and are available in the market: Benznidazole (BZL) and Nifurtimox (NFX)^3^. However, they have high adverse effects, intrinsic toxicity, and do not guarantee parasite eradication^3^. Additionally, the presence of natural BZL- and NFX-resistant strains in some CD patients remains an important issue, reducing the efficacy of these treatments^11^. Emerging trypanocidal medications such as Posaconazole and Ravuconazole, have shown adequate safety profiles and high efficacies, both in vitro and in vivo^12^ ^,^ ^13^. 

Combined therapy is a rational approach with the potential to increase clinical efficacy, decrease toxicity (by dose reduction), and decrease the likelihood of resistance (by achieving a more effective parasite elimination)^14^ ^,^ ^15^. However, the development of new therapeutic approaches for humans is limited by both ethical aspects, and the delayed diagnosis and sporadic case appearance of acute CD. As such, animal models are of significant importance. To the best of our knowledge, the therapeutic efficacy of the BZL+POS combination in CD has only been evaluated a few times using murine models^14^ ^,^ ^16^ ^-^ ^18^. This study therefore aimed at comparing the efficacy of the BZL+POS combination with that of monotherapy with either, in the reduction of *T. Cruzi* parasitic load in acute phase CD, using an experimental murine model.

## METHODS

### Experimental Design

Nineteen *Wistar rats* (Rattus norvergicus) with a median age of 40±2 days, and mean weight of 101±7 grams, were obtained from the Universidad Industrial de Santander (UIS). They were kept in an aired rack of the *Individually Ventilate Cages* (IVC-Tecniplast®, United Kingdom) system in a conventional facility with light/dark cycles (12/12), food (LabDiet 5001, Lab Supply®, United States of America), *ad libitum* water, temperature of 22±2°C, and relative humidity of 76%. The rats were observed and monitored daily. 

### Infection Model

The *T. cruzi’s* JChVcl1 strain, molecularly characterized as TcI, isolated from an acute CD patient, was used. It was isolated by the Grupo de Inmunología y Epidemiología Molecular (GIEM) at Universidad Industrial de Santander (UIS). The infectious forms (metacyclic trypomastigotes) were obtained via epimastigote incubation in the TAU3AAG medium. Rats were inoculated via intraperitoneal injection with 5x10^5^ trypomastigotes in a volume of 300μl. Intraperitoneal injection was performed in accordance with the international recommendation for substance administration^19^.

### Pharmacological treatment

Post infection, the rats were randomly allocated to four intervention groups. Group 1 (n=5) received BZL (Rochagan®, Hoffmann-La Roche, Basel, Switzerland) + POS (Noxafil®, MSD, NJ, USA) (100 mg/kg day + 20 mg/kg day); Group 2 (n=4), BZL (100 mg/kg day); Group 3 (n=5), NFX (LAMPIT®, Bayer AG, Leverkusen, Germany) (100 mg/kg day); and Group 0 (n=4) was untreated (control). The anti-parasitic therapy was administered from day 20-29 post-infection. All drugs were suspended in *ad libitum water,* and each animal received 0.2 ml of drug suspension by gavage with a syringe. In the control group, only the vehicle (ad libitum water) was administered.

### Parasitemia

Parasitemia quantification commenced on day 7 post-infection, and was registered every 2 or 3 days, until day 29. This was done using the Pizzi & Brener method: parasite counting in blood samples collected from tail vein punctures into microhematocrit-heparinized test tubes^20^. 

### Histopathological analysis

The animals were euthanized on day 33 post-infection using a CO_2_ chamber, in accordance with research and animal well-being regulations^21^. The samples were analyzed by an expert pathologist, unaware of the treatment group of all animals. Heart, colon, and skeletal muscle tissue samples were obtained. The animal hearts were split and perfused with an oxygenated Krebs-Henseleit solution, containing 50 mmol/L 2, 3 butanodione monoxime in a Langendorff model, at up to 37ºC. The hearts were then fixed in a neutral ink pad solution of up to 10% formalin, cut obliquely to an interventricular level, absorbed in paraffin, divided to 5 mm slices, and dyed with ammonia.

### Statistical analyses

Continuous variables are reported as mean and standard deviation, unless they did not present a normal distribution. Categorical variables are expressed as absolute or relative frequencies. An exact Fisher test was performed in order to evaluate differences between the quantity and nature of tissue infiltration. Kruskal-Wallis and Bonferroni tests were carried out to compare the median of the parasite load among the groups. 

Additionally, longitudinal data analysis for continuous outcome of repeated measures was performed using a generalized estimation equation (GEE) model^22^ ^-^ ^24^, considering the time after treatment commencement in days as a linear variable, parasitemia was chosen as the outcome and was measured at 20, 22, 24, 27 and 29 days of follow-up. Moreover, a compound symmetric (exchangeable) matrix was used to capture the correlations among repeated parasitemia measurements. A dummy variable of the treatment group was used as a covariate using a generalized estimation equation model (GEE) with a compound symmetric matrix. Lowes graph was used to compare parasitemia changes over time in the different treatment groups. A rat belonging to group 1 (G=1) was eliminated from the statistical analysis because it did not present with parasitemia. A *p-*value ˂0.05 was considered statistically significant. All statistical tests were two-sided. All data were analyzed using Stata Statistical Software: Release 14. College Station, TX: StataCorp LP.

### Ethical aspects of animal experimentation

This study was approved by the Ethics Committee of the Heart Institute, Floridablanca, under act No. 50 May 2012. All procedures were performed in accordance with resolution 8430 of the Health and Social Protection Ministry, Colombia, in which scientific, technical, and administrative regulations are established for investigations in health and law^25^ ^-^ ^26^.

## RESULTS

### Features of infection

**FIGURE 1:**
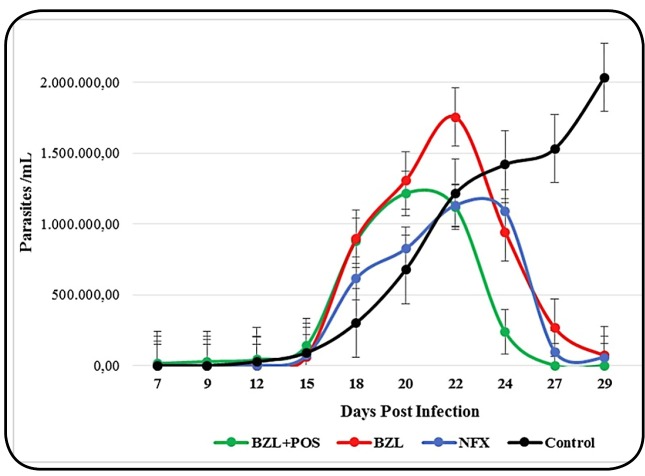
Parasitemia median tendency over time in the different treatment groups . Group 1 (n=5) received BZL+POS (100 mg/kg day + 20 mg/kg day); Group 2 (n=4), BZL (100 mg/kg day); Group 3 (n=5), NFX (100 mg/kg day); and Group 0 (n=4) was untreated (control). **BZL:** Beznidazol; **BZL+POS:** Beznidazol+Posaconazol; **NFX:** Nifurtimox; **Control:** non-treated.

At days 7, 10, and 11, animals with parasites were registered in one of the four treatment groups; however, infection was only noticeable from post-infection day 15. Likewise, the highest parasitemia variability was registered between days 15 and 27 (Figure 1). Parasitemia peaks for the three treatment groups were found (BZL, BZL+POS, and NFX) on post-infection days 20, 22, and 22, respectively, but not for the control group (day 29). 

### Treatment efficacy

At the commencement of the anti-parasitic treatment (post-infection day 20), there were no significant differences in the parasitemia medians of the treatment groups (BZL+POS=1.216.344, BZL=1.307.220, NFX=824.876, Control=678.076 parasites/mL; p=0.143). On post-treatment day 2 (post-infection day 22), parasitemia continued to increase in all but the BZL+POS group, which showed a median reduction of 97.867 parasites/mL; however, this difference statistically insignificant (p=0.156). On post-treatment day 4, statistically significant differences were observed in the parasitemia medians between BZL+POS=237.676 and BZL=943.715 (p=0.043) BZL+POS=237.676 and NFX=1.090.515 (p=0.032), and BZL+POS=237.676 and Control=1.419.068 parasites/mL (p=0.019). On post-treatment days 7 and 9, only the BZL+POS group achieved a median of 0.0 parasites/mL, which was statistically different from the control group (Table 1). 

**TABLE 1:** Treatment group and post-treatment day parasitic load.

	BZL+POS	BZL	NFX	Control	*p-	Comparison by	
Day	(n=5)	(n=4)	(n=5)	(n=4)	value	Group	†p-value
0	1.216.344	1.307.220	824.876	678.076	0.143	G1vsG2:	1.000;
	(992.648;	(1.132.458;	(615.162;	(580.210;		G1vsG3:	1.000;
	1.481.982)	1.363.144)	992.648)	824.876)		G1vsG0:	0.272;
						G2vsG0:	0.101;
						G3vsG0:	1.000;
						G2vsG3:	0.450.
2	1.118.477	1.754.611	1.132.458	1.216.344	0.156	G1vsG2:	0.076;
	(964.686;	(1.426.058;	(1.020.610;	(1.083.525;		G1vsG3:	1.000;
	1.188.382)	2.775.221)	2.013.259)	1.083.525)		G1vsG0:	1.000;
						G2vsG0:	0.358;
						G3vsG0:	1.000;
						G2vsG3:	0.416.
4	237.676	943.715	1.090.515	1.419.068	**0.015**	G1vsG2:	**0.043**;
	(209.714;	(664.095 -	(1.048.572;	(740.991;		G1vsG3:	**0.032**;
	265.638)	1.964.325)	1.342.173)	2.104.135)		G1vsG0:	**0.019**;
						G2vsG0:	1.000;
						G3vsG0;	1.000;
						G2vsG3:	1.000.
7	0	265.638	97.866	1.530.916	**0.016**	G1vsG2:	1.000;
	(0; 83.885)	(20.971 - 503.314)	(55.923; 167.771)	(1.300.230;		G1vsG3:	0.926;
				2.320.840)		G1vsG0:	**0.005**;
						G2vsG0:	0.096;
						G3vsG0:	0.089;
						G2vsG3:	1.000.
9	0	69.904	55.923	2.034.230	**0.007**	G1vsG2:	0.742;
	(0; 0)	(0 - 181.752)	(0 - 139.809)	(1.852.478 -		G1vsG3:	0.650;
				2.516.574)		G1vsG0:	**0.001**;
						G2vsG0:	0.092;
						G3vsG0:	0.069;
						G2vsG3:	1.000.

Medians (first and third quartil). **G1**=BZL+POS; **G2**=BZL; **G3**=NFX; **G0**=Control; **Values in bold** = p-value <0.05 of *Kruskal Wallis and †Bonferroni test. **BZL+POS=**Beznidazole+Posaconazole, **BZL=**Beznidazole; **NFX=**Nifurtimox; **Control:** non-treated.

### Longitudinal data analysis for parasitemia of repeated measures

In the marginal model fitted for days of treatment, the BZL+POS group achieved the greatest mean parasitemia reduction during the 9-day follow-up (β= -961.610 parasites/mL; CI 95%: -1.265.916, -657.305; p=0.000), followed by the NFX group (β= -687.583 parasites/mL; CI 95%: -991.888, -383.278; p=0.000), and then the BZL group (β= -457.876 parasites/mL; CI 95%: -778.642, -137.110; p=0.005) (Table 2). Figure 1 shows that the combined therapy group exerted its effect almost immediately after treatment commencement. Likewise, on post-treatment days 2 and 7, significant differences were observed in the parasitemia slopes of the BZL+POS group, compared with the BZL, NFX, and control groups.

**TABLE 2:** Efficacy of the different treatments in a repeated measurement analysis of parasitemia load (n=90).

Parasitemia	β	Standard Error	95% IC	p-value
**Time** (Days)*	-84.656	23.324	-130.372; -38.940	0.000
**BZL+POS**	-961.610	155.260	-1.265.916; -657.305	0.000
**BZL**	-457.876	163.659	-778.642; -137.110	0.005
**NFX**	-687.583	155.260	-991.888; -383.278	0.000
**Constant**	3.526.633	580.775	2.388.334; 4.664.933	0.000

**BZL+POS:** Beznidazole+Posaconazole; **BZL:** Beznidazole; **NFX:** Nifurtimox. Control group, reference; *variable time in days in the regression model of repeated measures.

### Histopathological findings

**FIGURE 2:**
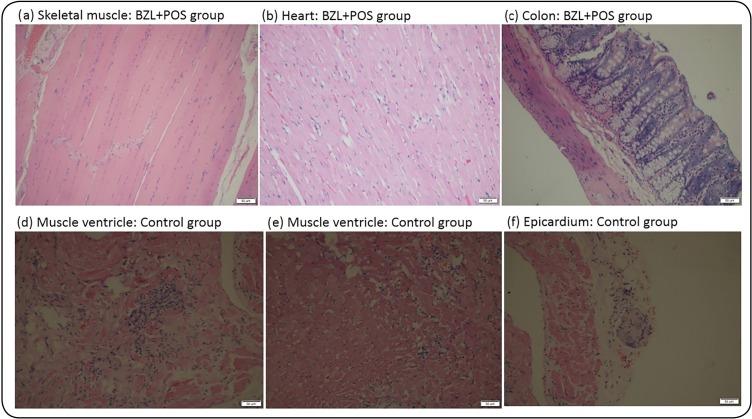
Histopathological findings in Wistar rat tissues. In panels, (**a), (b)** and **(c)**, skeletal, tight cardiac, and colon muscles were observed, respectively, with normal histological features. In the lower panel, (**d)** ventricular myocardium with added lymphocitary with measured fibrosis around it; (**e)** ventricular myocardium with edema represented by the division of muscle fibers and limited lymphocitary infiltrates in the interstice; and (**f)** epicardium with autonomous nerve ganglia infiltrated by lymphocytes. **BZL+POS:** Beznidazol+Posaconazol**; BZL:** Beznidazol; **NFX:** Nifurtimox; **Control**: non-treated.

Heart, skeletal muscle, and colon samples from 9 rats (3, each treatment group and 4, control group; total, 13 samples), were analyzed. The magnitude of visualized infiltrates was classified as non-presence, limited, moderate, or abundant, while the infiltrates were described as focal-interstitial, diffuse-interstitial, diffuse, or focal (Figure 2). Infiltrates were absent in all colon samples (100%; n=13), and present in one musculoskeletal sample. For heart samples, infiltrates were absent in the BZL+POS group; however, limited infiltrates were found in all BZL and NFX group samples. The differences between therapy schemes were statistically significant (p=0.001 Fisher’s test). In the analysis of the infiltrate types, statistically significant differences were found between treatment groups (p=0.013, Fisher’s test), none of the samples belonging to BZL+POS group had infiltrates, while 100% (n=3) and 67% (n=2) of the NFX and BZL group samples, respectively, presented with focal-interstitial infiltrates. Nests and amastigotes forms were absent in all analyzed samples. 

## DISCUSSION

This study is one of the first to use Wistar rat models to study CD treatment; hence, the behavior, pharmacokinetic characteristics, and pharmacodynamics of CD drugs in this animal remain unknown. However, the pharmacokinetic behavior of these drugs observed in murine models can be extrapolated to that found in Wistar rats, justifying the doses used in the Wistar rat models in the present study. In addition, the authors of this article previously validated the present model of infection and pharmacological treatment. The results of the present study show that at an optimal dose (100 mg/kg day + 20 mg/kg day), the BZL+POS combination therapy, can more efficaciously reduce parasite load than the NFX and BZL monotherapies in a *Wistar* rat model*.* Additionally, it appears that trypanocidal action begins earlier with the combination therapy, compared with NFX and BLZ monotherapies. 

Anti-parasitic treatment during acute phase CD is the central pillar of disease management, partially because of the higher parasite load in comparison with the chronic phase^27^ ^-^ ^29^, in which the etiological treatment has not shown beneficial against cardiopathological progression^30^. Currently, BZL and NFX are the treatment of choice for CD, with BZL being more widely used, owing to its excellent pharmacological efficacy and less adverse effects^31^ ^-^ ^33^. Cancado et al.^34^ found differences in the proportion of recovered patients treated with BZL for extended periods according to the CD phase; 76% in the acute phase compared with just 8% in the chronic phase. Additionally, evidence suggests that treatment during this phase could prevent CD progression to the indeterminate or chronic stage^31^ ^,^ ^35^. The limited efficacy of BZL during the chronic phase is probably owed to its relatively shorter half-life and limited tissue penetration, restricting its action in parasite confinement to deep tissues^35^ ^-^ ^37^. 

In this study, we found that BZL monotherapy (100 mg/kg day) was associated with lower parasitemia reduction, compared with the other treatments. The use of BZL at a dose of 100 mg/kg/day in mouse models^38^ ^-^ ^39^ could be extrapolated to Wistar rat models, justifying the use of this dose in our murine model, but not the low trypanocidal capacity found in this study. Some studies in murine models have shown that low BZL doses (25 mg/kg/day), compared to those used in other studies (BZL 100 mg/kg/day), are more useful for parasite elimination^40^. Contrarily, in a study in BALB/c mice, the 100 mg/kg/day optimal dose was found to be curative^41^.

Given the possibility of parasitic resistance, potential adverse effects, and low clinical efficacy during the chronic phase, novel therapeutic approaches have been proposed based on new biochemical pathways, including the blocking of sterol biosynthesis, and cysteine protease and pyrophosphate metabolism inhibitors, with promising pre-clinical study results^35^.

POS is one of the most relevant molecules in this search for new CD treatments. Some studies suggest that the activity of POS could be similar or even superior to that of BZL in acute CD treatment in murine models^42^. However, subsequent vitro and in vivo studies have shown opposite results^43^ ^,^ ^44^ ^,^ ^45^ when comparing monotherapy with both drugs. These findings could be explained by the fact that the achieved human drug systemic concentrations at the dose used in these studies (400 mg twice a day) are just 10-20% of that achieved in mice at the minimum curative dose (20 mg/kg/day)^46^ ^,^ ^47^.

However, the BZL+POS combination therapy appears to be a more promising CD treatment, since theoretically, it should improve treatment efficacy by acting on different cellular targets and metabolic pathways, with the potential advantage of minimizing the resistance risk^16^ ^,^ ^17^ ^,^ ^48^ ^,^ ^49^. Additional benefits of this approach include BZL dose reduction and shorter treatment schemes^16^; since this study did not include groups with lower doses of BZL alone or a BZL + POS combination than those currently used (100 mg/kg day + 20 mg/kg/day), we cannot conclude on whether treatment with a lower dose of the combination therapy would have the same efficacy as treatment with the full dose.

Bustamante et al^17^ found that treatment of BZL resistant strain-infected mice with POS (100 mg/kg/day) once every five days, or with a combination of five daily doses and 7 sporadic doses of BZL (20 mg/kg/day), provided a 100% recovery rate. Likewise, Diniz et al.^18^ described a similar positive synergistic effect in a murine model administered BZL and POS individually or in combination (25, 50, 75, or 100 mg/kg/day BZL in combination with 5, 10, or 20 mg/Kg/day POS); the combination therapy was reportedly more active, with regards to parasitemia reduction, compared to monotherapy. Accordingly, treatment strategies involving lower administration time and doses of the BZL monotherapy or the BZL+POS combination therapy, compared to those of current therapies, could help minimize toxicity.

Combinations of BZL and other molecules have also been evaluated. Batista et al*.* ^16^ showed that the BZL+DB289 combination reduced both parasitemia (99%) and mortality, but did not provide a successful parasitological recovery. Additionally, the BZL+DB766 combination reduced parasitemia (at least 99.5%) and tissue damage via a 9-fold increase in trypanocidal activity, compared to BZL monotherapy.

Other studies have evaluated the pharmacokinetics of combination with BZL and other azoles, and monotherapy with BZL, in murine models^48^ ^,^ ^50^. The administration of BZL+Itraconazole decreased (1.5 times) maximum drug concentration in plasma, and increased (2.66) distribution volume and BZL half-life (7.7 times). The effect was similar with BZL+Ketaconazole administration to Swiss mice^50^. In this model, a higher proportion of mice recovered in the combined therapy than the monotherapy group. This positive synergistic effect could be one of the primary mechanisms of the findings of the present study. 

Contrary to this synergistic evidence and the findings of the present study, Cencig et al^14^. reported that the recovery rate of mice infected with strain *Y* was not improved by the BZL+POS combination therapy, compared with the POS monotherapy. It is necessary to consider that the synergistic effect of the combination therapy in some studies could vary between endemic regions, given the percentage of resistance to monotherapy with BZL recently described for some strains^51^. 

On the other hand, other studies in murine models have shown that the efficacy of the treatment scheme could considerably vary depending on the anti-parasitic dose, treatment days, and immune response of the rodent^43^ ^,^ ^45^ Regarding the host immune response, several studies have shown that cellular activation of the immune system by IL-12 favors BZL activity against *T. cruzi,* and that the efficacy of monotherapy with BZL or POS could vary if the rodent has deficiencies or over-expresses some immune response expanders such as IL-12 and INF-gamma, and other inflammatory mediators^11^ ^,^ ^27^ ^,^ ^42^ ^,^ ^52^. 

Another important finding in our study is the lower rate of histological lesions in the myocardial tissue of combination therapy-treated animals, suggesting that early parasite elimination is associated with lesser tissue involvement^53^ ^-^ ^55^.

### Strengths and limitations

This is, to the best of our knowledge, the first study in Colombia wherein a murine model is used to evaluate the efficacy of the BZL+POS combination therapy in *T*. *cruzi* parasitic load reduction in acute phase CD. Another advantage of this study is the random allocation of treatment schemes, and the use of a longitudinal model of repeated measurements to gain statistical power and for post-treatment anti-parasitic effect evaluation. 

One of the most critical limitations of this study is the probable introduction of bias by the parasitologist, resulting from their awareness of the treatment groups. Others include the low number of animals (n=5) in the experimental groups, drug optimal dose determination from previous studies in mice (as such doses have not been optimized for rats), and lack of model adjustment for important variables such as weight and animal age, which could impact parasitic load. However, we ensured that all observed groups were under the same experimental conditions throughout the study. 

Other relevant limitations include the sub-optimal method used for parasitemia evaluation (using classical light microscopy methods instead of T. cruzi-DNA PCR assays) and the non-uniform intervals for parasitemia measurement. Moreover, after treatment day 29, parasitemia was not measured, preventing the evaluation of post-treatment parasitemia behavior. In addition, monotherapy with 20 mg/kg Posaconazole or combination therapy with NFX/POS was not performed, preventing the evaluation of the efficacy of 20 mg/kg Posaconazole and NFX/POS, compared to the other treatments. Likewise, we failed to compare the effects of the drugs administered alone and in combination, at optimal and suboptimal doses, on efficacy and toxicity, to ascertain if the combined effects may be synergistic, additive, or antagonistic. Finally, for the histological analysis, only 13 samples from the 19 rats included in the study were analyzed, introducing bias.

## CONCLUSIONS

The BZL+POS combination therapy was most efficacious against acute phase CD, with regards to parasitic load and parasite infection-induced myocardium lesion reduction, compared to the NFX and BZL monotherapies, in the *Wistar* rat models*.* Additionally, BZL+POS reduced parasite load almost immediately after treatment commencement, compared to the other therapies. Further, the BZL+POS combination might be a potential therapy for acute phase CD patients in need of aggressive parasite control or for whom resistance is suspected, like immunocompromised patients.
